# Evaluation of the Usefulness of YouTube Videos on Retinal Detachment Surgery

**DOI:** 10.7759/cureus.19457

**Published:** 2021-11-10

**Authors:** Murat Serkan Songur, Mehmet Citirik

**Affiliations:** 1 Ophthalmology, Bozok University Faculty of Medicine, Yozgat, TUR; 2 Ophthalmology, Ulucanlar Eye Education and Research Hospital, Ankara, TUR

**Keywords:** global quality score, jama score, discern score, youtube, retinal detachment surgery

## Abstract

Introduction

The aim of this study is to evaluate the usefulness of YouTube videos about retinal detachment surgery as a resource.

Methods

The first 100 videos were evaluated when they were scanned by typing "retinal detachment surgery " in the YouTube search engine. These videos were also analyzed and scored using DISCERN, Journal of the American Medical Association (JAMA), and Global Quality (GQ) scoring systems.

Results

The DISCERN score of the evaluated videos was 39.5±8.4; JAMA score was 1.9±0.5; and the GQ score was 2.1±0.5. According to the results, retinal detachment surgery videos, DISCERN score is medium; The JAMA score was evaluated as low quality and poor quality in the GQ score.

Conclusion

Although there are enough videos on YouTube with retinal detachment surgery, its usefulness as a resource is low, and its quality is poor.

## Introduction

Retinal detachment is the separation of the neurosensory retina (NSR) from the underlying retinal pigment epithelium (RPE) [1]. Although the pathogenesis of retinal detachment is not completely understood, symptomatic retinal tear is often associated with vitreous traction and carries a great risk for retinal detachment [2]. The most common type of retinal detachment is rhegmatogenous retinal detachment. The incidence of retinal detachment is also increasing after cataract surgery [3]. In the treatment of retinal detachment, scleral buckling, pars plana vitrectomy (PPV), pneumatic retinopexy, and combination techniques are used [4]. Air, sulfur hexafluoride (SF6) gas, perfluoropropane (C3F8) gas, and intravitreal silicone tamponade can be used in patients with retinal detachment treated with PPV [5].

YouTube has become one of the most popular websites in the world, with over two billion users and over 500 hours of video content uploaded to the website every minute [6]. The internet has provided the opportunity to access almost all necessary information easily and free of charge. The increasing availability of video sharing sites, such as YouTube, has led to their use as a tool for obtaining information in the field of health as well as in other fields. However, using YouTube as a source of information in the field of health can cause various problems. Problems such as uploading videos by non-healthcare professionals, presentation of opinions without sufficient knowledge and experience, videos used for advertising purposes, lack of detailed information about the contraindications and complications of an operation or procedure, and the lack of a controlling evaluation process occur. In addition, patients and individuals with incomplete medical knowledge may evaluate health-related information from their own perspective, which may lead to the dissemination of incomplete or inaccurate information [7].

Our aim in this study is to evaluate retinal detachment surgery videos that have not been evaluated before in the literature and to provide an overview by evaluating the quality and educational contribution of these videos through scoring.

## Materials and methods

This study was conducted in September 2021 by retrospectively viewing videos that were publicly available on YouTube. The principles of the Declaration of Helsinki were adhered to during the study. We evaluated the first 100 videos by typing "retinal detachment surgery" in the YouTube search engine. These videos were recorded in terms of duration, likes and dislikes, release date, content, and the number of views. Only videos in English were taken into consideration.

The videos were independently evaluated by the two authors, who are experienced ophthalmologists. All videos were independently scored by DISCERN, Journal of the American Medical Association (JAMA), and Global Quality (GQ), and the results were averaged.

There are 16 questions in total in DISCERN scoring. The score of all questions ranges from one to five. The first eight questions are used to determine the credibility of the web page. The second part, 9 through 15, evaluates the quality of information about treatment options. The 16th and final question is a general evaluation of the website. There is a score between 16 and 75 in DISCERN scoring. In DISCERN scoring, it is classified as very poor between 16 and 26 points, poor between 27 and 38, moderate between 39 and 50, good between 51 and 62, and excellent between 63 and 75 [8]. DISCERN questions are shown in Table 1.

**Table 1:** Questions in the DISCERN scoring system and the Global Quality (GQ) scoring system

Questions in the DISCERN scoring system	
SECTION 1	
Question 1	Are the goals clear?
Question 2	Does it reach its goals?
Question 3	Is it relevant?
Question 4	Are the publication sources used to compile information compatible?
Question 5	Is it clear when information is used or reported?
Question 6	Is it balanced and unbiased?
Question 7	Does it provide additional support resources and information?
Question 8	Does it refer to indefinite fields?
SECTION 2	
Question 9	Does it explain how each treatment works?
Question 10	Does it explain the benefits of each treatment?
Question 11	Does it explain the risks of each treatment?
Question 12	Does it explain what can happen if left untreated?
Question 13	Does it explain how much each treatment can affect quality of life?
Question 14	Does it explain that there may be more than one possible treatment choice?
Question 15	Does it provide support for joint decision making?
SECTION 3	
Question 16	What is the overall quality rating like?
Global Quality (GQ) scoring system	
1 - Bad quality	Not likely to be used for patient education
2 - Bad quality	Limited use for patients; because only some information is available
3 - Insufficient quality and flow	It is somewhat helpful; important topics are missing, some information is available
4 - Good quality and streaming	It is useful for patients; because the most important topics are covered
5 - Excellent quality and flow	Very useful for patients

JAMA criteria are used to evaluate basic information presented on websites. Basically, it includes authorship, bibliography, patent right, and timeliness. Each criterion gets one point. A one indicates the weakest quality and a four indicates the highest quality [9].

GQ scoring provides the opportunity to interpret the videos in general and to evaluate the overall quality of the videos according to the information flow presented [10]. In the GQ scoring, the scoring ranges from one to five. The GQ scoring system is shown in Table 1 [11].

All analyzes were performed using the SPSS Windows V.21.0 software package (IBM Inc., Armonk, New York). The mean SD was used for continuous data. Student T-test was used for pairwise comparison of data for normally distributed data. Spearman correlation test was applied to examine the relationships between the variables. A p-value less than 0.05 was considered significant.

## Results

In our study, 87 videos that met the inclusion criteria out of a total of 100 videos that emerged as a result of searching for the keyword in the YouTube search engine were analyzed and evaluated. Of these videos, three were excluded because they were irrelevant, three were in a language other than English, and seven were excluded because of the content of retinal diseases and surgery other than retinal detachment surgery. The general features of the videos are summarized in Table 2.

**Table 2:** General features of videos and comparison of videos with and without surgery

General features of videos			
Broadcast time (seconds)		805.5	
Number of likes		169.5	
Number of dislikes		7.5	
Broadcast history (month)		39.2	
Views		27114.5	
Comparison of videos with and without surgery			
	Videos containing surgery (n=58)	Non-surgical videos (n=26)	p -value
Video duration (sec)	874.5±125.6	728.2±168.6	0.646
Number of likes	64.2±14.4	287.7±50.0	0.005
Number of dislikes	3.7±1.2	11.7±2.1	0.036
Views	12689.0±449.1	43299.3±618.5	0.009
DISCERN score	41.5±5.6	37.2±10.3	0.016
JAMA score	2.1±0.4	1.7±0.5	0.001
Global Quality score	2.2±0.4	1.9±0.6	0.008

None of the evaluated videos contained advertising content. All of the videos were uploaded by ophthalmologists. There was a significant correlation between the viewing rates of all videos and the number of likes (p<0.001; r=0.850) and dislikes (p=<0.001; r=0.817). No correlation was found between the duration of the videos and the rate of viewing (p=0.978; r=0.273). In the videos, 46 videos were surgical, while 41 videos did not have a surgical presentation. There was no statistically significant difference between the duration of the videos with and without surgery. The number of likes and dislikes and the number of views of the non-surgical videos were statistically significantly higher than the surgical videos. However, videos with surgical content were found to have statistically significantly higher quality in DISCERN (p=0.016), JAMA (p=0.001), and GQ (p=0.008) scores than videos without surgical content (Table 2).

The DISCERN score of the evaluated videos was 39.5±8.4; JAMA score was 1.9±0.5; and the GQ score was 2.1±0.5. According to the results, retinal detachment surgery videos, DISCERN score was moderate; The JAMA score was assessed as low quality and the GQ scoring as poor quality.

## Discussion

In our study, we found that in videos on retinal detachment surgery on YouTube, the DISCERN score was moderate, the JAMA and GQ scores were low, and the videos were of insufficient quality. We also found that all three scores were higher in videos with surgical content than those without surgical content.

YouTube is the world's largest media-sharing website and the second most used website worldwide. In addition to providing a platform for information and moral support, the internet has created opportunities for open discussion about health and medicine. Unfortunately, this increased opportunity has led to the increase and dissemination of false and even harmful information. The rise in published studies on YouTube video reliability is proof of this [12].

Borgersen et al., in their study, determined that only 27 of 7640 videos were suitable for use in the YouTube video search for direct ophthalmoscope [13]. Benetoli et al. revealed that many social media users turn to health-related YouTube videos for emotional support, especially during a chronic illness. However, they stated that since many people do not trust these videos, they still want to meet with doctors face-to-face for information [14]. Sahin et al. found that one-third of the videos on retinopathy of prematurity on YouTube were misleading and could have harmful consequences [15]. Abdelmseih et al. examined the quality of YouTube videos in age-related macular degeneration. Of the videos they reviewed, 60% were rated as partially applicable, 35% as misleading, and 5% as irrelevant. According to reliability, 60% of videos are classified as partially reliable, 35% as unreliable, and only 5% as reliable [16].

Aykut et al., in their study where they examined YouTube videos of cataract surgeries performed on eyes with small pupils, found that only one of the videos had significant complications and one had minor complications. However, considering that the complication rate is normally higher in eyes with small pupils, concluded that such videos are published less frequently [17]. Similarly, no complications were found in any of the videos in our study.

Nicholl et al. reported that parents preferred accurate information (65%), reliable information (62%), and updated information (61%) in information research [18]. Since this shows especially DISCERN and JAMA scoring, we think that it may be appropriate to use these scorings in video evaluation. In their YouTube study of contact lenses, Yıldız et al. found the videos have an average DISCERN score of videos to be 32.3, JAMA score to be 1.2, and GQ score to be 3.4. They stated that the video quality is higher in videos published by universities and professional organizations [19]. In Küçük et al.'s review of refractive surgery videos on YouTube, the average DISCERN score of the videos was 33.2; JAMA score of 0.7; they found that the GQ score was 1.7 and as a result, the video quality was poor [20]. In our study, the mean DISCERN score was 39.5; The JAMA score was 1.9, and the GQ score was 2.1 and the videos were generally of poor quality. As far as we know, there is no scientific article examining retinal detachment surgery videos on YouTube.

Online videos for patient education and the issues with their quality and accuracy have received more attention recently. Social media has great potential to provide easy access to medical information, but it is not possible to ensure that the information received is accurate and unbiased.

Our study has several limitations. First of all, we do not have enough information about the pre- and post-operative videos that we consider surgical. Secondly, we evaluated videos in English. Third, the video evaluation was subjective, although it was evaluated independently by two experienced surgeons. Further studies are needed to evaluate youtube videos in retinal detachment.

As a result of this study, YouTube videos labeled "retinal detachment surgery" often contain poor content quality and incomplete information. In order for these videos to be used as a source of information, they should be recorded by more qualified professionals, presenting their content and all information about all treatment options, complications, and healing processes in an objective way.

## Conclusions

As a result, according to our findings, YouTube videos labeled "retinal detachment surgery" often contain poor content quality and incomplete information. In order for these videos to be used as a source of information, they should be recorded by more qualified professionals, presenting their content and all information about all treatment options, complications, and healing processes in an objective way.
